# Genetic and epigenetic variation in *Spartina alterniflora* following the *Deepwater Horizon* oil spill

**DOI:** 10.1111/eva.12482

**Published:** 2017-05-12

**Authors:** Marta Robertson, Aaron Schrey, Ashley Shayter, Christina J Moss, Christina Richards

**Affiliations:** ^1^ Department of Integrative Biology University of South Florida Tampa FL USA; ^2^ Department of Biology Armstrong State University Savannah GA USA; ^3^ Rehabilitation Institute Southern Illinois University Carbondale IL USA; ^4^ Department of Cell Biology Microbiology and Molecular Biology University of South Florida Tampa FL USA

**Keywords:** AFLP, *Deepwater Horizon*, DNA methylation, environmental stressors, epigenetics, MS‐AFLP, *Spartina alterniflora*

## Abstract

Catastrophic events offer unique opportunities to study rapid population response to stress in natural settings. In concert with genetic variation, epigenetic mechanisms may allow populations to persist through severe environmental challenges. In 2010, the *Deepwater Horizon* oil spill devastated large portions of the coastline along the Gulf of Mexico. However, the foundational salt marsh grass, *Spartina alterniflora*, showed high resilience to this strong environmental disturbance. Following the spill, we simultaneously examined the genetic and epigenetic structure of recovering populations of *S. alterniflora* to oil exposure. We quantified genetic and DNA methylation variation using amplified fragment length polymorphism and methylation sensitive fragment length polymorphism (MS‐AFLP) to test the hypothesis that response to oil exposure in *S. alterniflora* resulted in genetically and epigenetically based population differentiation. We found high genetic and epigenetic variation within and among sites and found significant genetic differentiation between contaminated and uncontaminated sites, which may reflect nonrandom mortality in response to oil exposure. Additionally, despite a lack of genomewide patterns in DNA methylation between contaminated and uncontaminated sites, we found five MS‐AFLP loci (12% of polymorphic MS‐AFLP loci) that were correlated with oil exposure. Overall, our findings support genetically based differentiation correlated with exposure to the oil spill in this system, but also suggest a potential role for epigenetic mechanisms in population differentiation.

## 1 INTRODUCTION

Ecological theory predicts that adaptation to local conditions can result when populations harbor heritable phenotypic variation for traits that increase tolerance to local conditions. Classic population genetics studies demonstrate that natural selection in different microhabitats can result in associations of genotypes, or alleles of candidate genes, with habitat type (e.g., Hamrick & Allard, 1972; Salzman, 1985; Schmidt & Rand, 1999; Schmidt et al., 2008). In concert with other evolutionary mechanisms, disturbance events may also create population genetic structure, by diminishing standing genetic diversity through mortality (Hermisson & Pennings, 2005; Orr & Betancourt, 2001). These classic predictions are intuitive and often supported empirically (e.g., Clausen, Keck, & Hiesey, 1948). However, in some cases, data across a diversity of taxa show either no association of genetic differences with habitat (e.g., Richards, Hamrick, Donovan, & Mauricio, 2004; Foust et al., 2016; examples in Schmidt et al., 2008) or that low levels of molecular diversity are not associated with decreased phenotypic variation (Dlugosch & Parker, 2008; Richards et al., 2008). The disconnect between empirical findings and ecological theory suggests the possibility of additional, underexplored molecular mechanisms, such as epigenetic modifications, that mediate the relationship between phenotype and environment.

The recent application of molecular techniques to ecological questions has revealed that epigenetic regulatory mechanisms, such as DNA methylation, may respond dynamically and independently to sudden changes in the environment (e.g., Gugger, Fitz‐Gibbon, Pellegrini, & Sork, 2016; Trucchi et al., 2016). Although there are several epigenetic mechanisms that can alter gene expression (e.g., chromatin remodeling, histone modifications, small interfering RNAs), DNA methylation of cytosines is the most widely studied (Schrey et al., 2013; Verhoeven, Vonholdt, & Sork, 2016) and can have important ecological effects. For example, studies in *Taraxacum officinale* show that when DNA methylation machinery is disrupted, flowering time differences among populations of these plants are removed (Wilschut, Oplaat, Snoek, Kirschner, & Verhoeven, 2016). Additionally, natural populations typically harbor high amounts of epigenetic variation (Keller, Lasky, & Yi, 2016; Paun et al., 2010; Richards, Schrey, & Pigliucci, 2012), which can be structured by local environmental conditions along with genetic variation. Variation in DNA methylation is correlated with habitat type in mangroves (Lira‐Medeiros et al., 2010) and knotweed (Richards et al., 2012), herbivory in viola (Herrera & Bazaga, 2010), and climate in natural accessions of *Arabidopsis thaliana* (Keller et al., 2016). This association between DNA methylation and plant ecology may reflect the modulation of gene expression (Bewick et al., 2016; Zilberman, Gehring, Tran, Ballinger, & Henikoff, 2007) or recombination rates (Mirouze et al., 2012), the release of transposable elements (Dowen et al., 2012), or other regulatory processes in response to environmental conditions in addition to covariance with genetic structure. In some cases, epigenetic variation can be restructured during periods of environmental stress and these changes can persist after the stress is relieved (Verhoeven, Jansen, van Dijk, Biere, 2010; Verhoeven, Van Dijk, Biere, 2010; Dowen et al., 2012 but see Wibowo et al., 2016). These findings suggest that epigenetic mechanisms may allow for rapid modification of phenotype in response to immediate and acute stressors (Rapp & Wendel, 2005).

In this study, we simultaneously examined genetic and epigenetic patterns in populations of *S. alterniflora* along the Gulf Coast that were exposed to heavy oiling following the *Deepwater Horizon* (*DWH*) oil spill (“heavy” sensu Lin et al., 2016; Nixon et al., 2016). In 2010, 4.9 million barrels of oil spilled into the Gulf of Mexico over a period of 3 months, with devastating effects on coastal ecology and salt marsh ecosystems (Lin & Mendelssohn, 2012; Lin et al., 2016; Silliman et al., 2012; Whitehead et al., 2012). As the dominant plant on the leading edge of salt marshes, many *S. alterniflora* populations across the northern Gulf of Mexico were negatively impacted by the *DWH* oil spill. Despite large die‐off of aboveground biomass and reduced carbon fixation and transpiration in heavily oiled populations, *S. alterniflora* showed high resilience to the hydrocarbon exposure (Lin & Mendelssohn, 2012; Lin et al., 2016), and aboveground biomass and live stem density levels recovered to the same level as uncontaminated reference marshes within 18 months (Lin et al., 2016). However, while these and other studies support that *S. alterniflora* is resilient to hydrocarbon stress, the extent of intraspecific variation in resilience is uncertain, and it remains unknown whether there was differential mortality among *S. alterniflora* genotypes in natural populations exposed to the *DWH* oil spill. We measured genetic and epigenetic variation using amplified fragment length polymorphism (AFLP) and methylation sensitive fragment length polymorphism (MS‐AFLP) to test the hypothesis that oil exposure in *S. alterniflora* resulted in genetic and epigenetic signatures of population differentiation. As in previous studies of *S. alterniflora* (Edwards, Travis, & Proffitt, 2005; Foust et al., 2016; Hughes & Lotterhos, 2014; Richards et al., 2004; Travis, Proffitt, & Ritland, 2004), we expected to see high levels of genetic and epigenetic variation. However, we anticipated that moderate, nonrandom differential mortality in response to oil exposure would result in genetic differentiation of oil‐exposed populations from unexposed populations. Further, we anticipated a concurrent but stronger epigenetic signature of oil exposure, given its reflection of gene expression and physiological response to environmental stimuli (Dowen et al., 2012; Verhoeven, Jansen, et al., 2010; Verhoeven, Van Dijk, et al. 2010; Xie et al., 2015).

## 2 MATERIALS AND METHODS

### 2.1 Sample collection

*Spartina alterniflora* is a clonal halophyte, native to the east coast of the United States and invasive in coastlines around the world (Ainouche et al., 2009; Ayres, Smith, Zaremba, Klohr, & Strong, 2004; Pennings & Bertness, 2001). *Spartina alterniflora* displays diverse phenotypes in response to the natural environmental gradients in marshes, producing less aboveground biomass in response to increasingly saline soil (Richards, Pennings, & Donovan, 2005). Populations of *S. alterniflora* display high genetic diversity (Edwards et al., 2005; Foust et al., 2016; Hughes & Lotterhos, 2014; Richards et al., 2004; Travis et al., 2004) and substantial resilience to both natural variation in the salt marsh (Pennings & Bertness, 2001) and anthropogenic stressors, such as crude oil (Lin & Mendelssohn, 2012; Lin et al., 2016).

**Figure 1:**
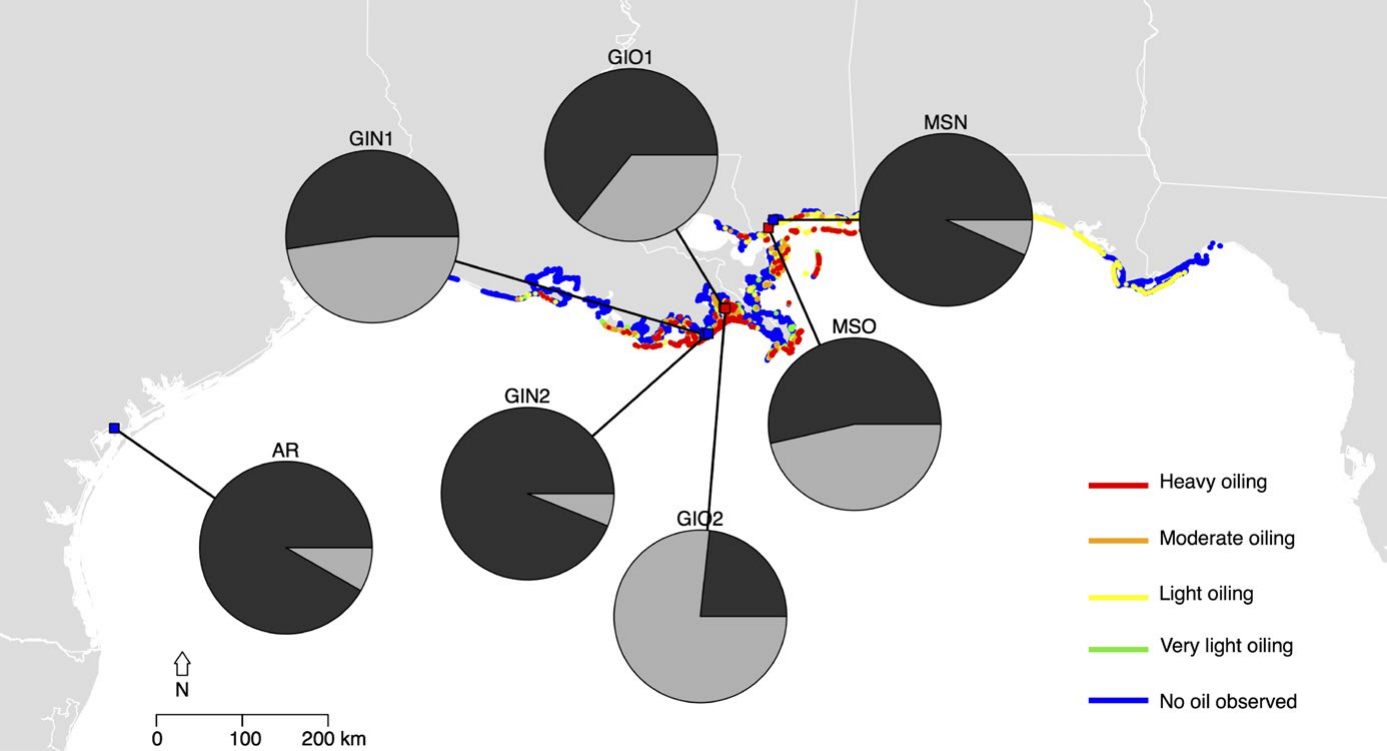
Map of seven study sites and their relative locations in the Gulf Coast, with site‐specific oil intensity following the *Deepwater Horizon* (*DWH*) oil spill, according to NRDA databases, and the results of Bayesian clustering. Population assignment to two groups is indicated by the shaded portion of the circle for each species. Group 1 = dark gray, group 2 = light gray

**Figure 2:**
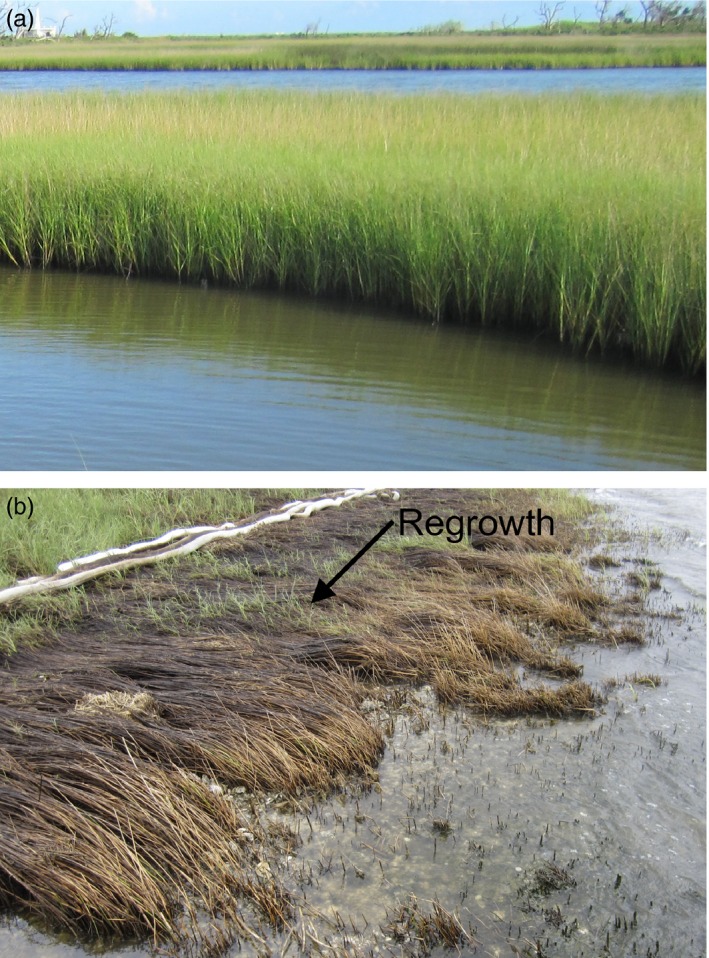
Examples of (a) noncontaminated Grand Isle, LA no‐oil site 1, (GIN1) and (b) contaminated sites Grand Isle, LA site 1 (GIO1) in the Gulf Coast following the *Deepwater Horizon* (DWH) oil spill. Oil was present on the soil surface at the time of sampling, and plants experienced substantial dieback. New growth sampled for this study (arrow) can be seen emerging from ramets under the soil surface through the dead wrack aboveground

We collected leaf tissue from *S. alterniflora* stems at approximately ten‐meter intervals along the shoreline from three oil‐contaminated and four uncontaminated reference sites along the Gulf Coast in August 2010, while oil was still standing on the soil surface at contaminated sites (Table 1; Figure 1). Oil contamination was defined by visually confirmed presence of crude oil in the sediment and complete aboveground dieback of *S. alterniflora* in populations on the leading edge of the marsh. The only visible live tissue was the regrowth of stems from rhizomes through the wrack of dead aboveground *S. alterniflora* (Figure 2) from which we collected leaf tissue. Contamination levels were later confirmed through Natural Resource Damage Assessment databases (2014; Figure 1). Shoreline Cleanup Assessment Technique categories delineate oil contamination into five categories from “no oil observed” to “heavy oiling” (Nixon et al., 2016; Zengel et al., 2016). Our three contaminated sites fit the description of heavily oiled marshes, whereas the four uncontaminated sites had no visible oiling or impacted vegetation at the time samples were collected, and were not annotated as contaminated in the oil assessment databases. Samples were collected from the middle of the so‐called tall plant zone near the leading edge of the marsh (sensu “low‐salt habitat” in Foust et al., 2016). From each plant, we collected the 3rd fully expanded leaf to standardize age and minimize developmental bias in sampling. The contaminated sites were Grand Isle, LA oiled site 1 (GIO1) (*n* = 6); Grand Isle, LA oiled site 2 (GIO2) (*n* = 7); and Bay St. Louis, MS oiled (MSO) (*n* = 8). Nearby uncontaminated reference sites were Grand Isle, LA no‐oil site 1 (GIN1) (*n* = 9), Grand Isle, LA no‐oil site 2 (GIN2) (*n* = 10), and Bay St. Louis, MS no‐oil (MSN) (*n* = 8). Because the minimum number of populations required to detect differences between two groups at the level of alpha = 0.05 is suggested to be *n* = 7 (Fitzpatrick, 2009), we also sampled one additional reference site, Aransas, TX (AR) (*n* = 10), which was not affected by the *DWH* oil spill (Table 1). Sites in Mississippi and Louisiana were separated by a minimum of 10 km and maximum of 35 km, and AR was 775 km from Mississippi. Tissue samples were snap‐frozen in liquid nitrogen and stored at −80°C.

**Table 1:** GPS coordinates of seven study sites

Population	Coordinates		
	*N*	Longitude	Latitude
Oil‐contaminated			
GIO1	6	29°26′42.8″N	89°55′45.7″W
GIO2	7	29°26′11.2″N	89°54′35.9″W
MSO	8	30°15′29.1″N	89°24′45.6″W
Unaffected			
GIN1	9	29°10′09.2″N	90°09′05.7″W
GIN2	8	29°10′49.4″N	90°06′31.6″W
MSN	8	30°20′21.1″N	89°21′15.3″W
AR	10	28°13′00.3″N	96°59′16.8″W

### 2.2 AFLP genotyping

We used AFLP to assess genetic variation between the field sites using a standard protocol described in Richards et al. (2012). Briefly, we isolated DNA in duplicate from leaf tissue with the Qiagen DNeasy Plant Mini Kit according to the manufacturer's recommended protocol (Qiagen, Valencia, CA) and conducted the entire protocol on duplicate reactions to ensure the consistent scoring of fragments and control for the potential error rate of AFLP markers. For selective PCR, we used fluorescently labeled primers *Eco*RI + AGC (6‐FAM) and +ACG (HEX) and unlabeled *Mse*I + CAC primers. We sent selective PCR products to the DNA Facility at Iowa State University, IA, USA, where they were electrophoresed on an ABI 3130XL. We scored resulting fragments in duplicate as “1” for present and “0” for absent using Peak Scanner (Thermo Fisher Scientific) and excluded markers that were not supported in duplicate.

### 2.3 MS‐AFLP epigenotyping

We used MS‐AFLP to assess genomewide DNA methylation on the same duplicate DNA extractions used in the AFLP protocol (Reyna‐Lopez, Simpson, & Ruiz‐Herrera, 1997). We used *MspI* and *HpaII* restriction enzymes, which have different sensitivities to cytosine methylation of the same CCGG sequence (Reyna‐Lopez et al., 1997; Salmon, Clotault, Jenczewski, Chable, & Manzanares‐Dauleux, 2008). DNA extracts were digested with both *EcoRI/MspI* and *EcoRI/HpaII* enzyme combinations independently for each individual, and selective PCR was run with fluorescently labeled primers *EcoRI *+ AGC (6‐FAM) and +ACG (HEX) and unlabeled primers *HpaII/MspI* +TCAC and *HpaII/MspI *+ TCAT. We sent selective PCR products to the DNA Facility at Iowa State University, IA, USA, where they were analyzed on an ABI3130XL. We visualized the resulting electropherograms using Peak Scanner and scored fragments as “1” when present and “0” when absent.

Together, *MspI* and *HpaII* produce four types of evaluative variation (Salmon et al., 2008). *MspI* does not cut when the external cytosines are fully or hemimethylated, and *HpaII* does not cut when either the internal or external cytosines are methylated on both strands. Likewise, cleaving by both enzymes is blocked when both cytosines are methylated. The resulting fragments can be classified as either type I when the corresponding sequence restriction site is nonmethylated and fragments occur in both digests, type II when fragments are absent in *EcoRI* + *HpaII* digests but present in *MspI*, type III when fragments are absent in *EcoRI* + *MspI* digests only, or type IV when no fragments occur in either digest. We treated type IV variation as missing data, because the methylation state cannot be specified (Salmon et al., 2008). Although some advocate for discriminating between type II and type III methylation as these types are expected to capture methylation in CG versus CHG contexts (Medrano, Herrera, & Bazaga, 2014; Schulz, Eckstein, & Durka, 2014), type II variation and type III variation cannot simply be interpreted as CG versus CHG methylation as apparent CHG methylation can be caused by the nesting of internal restriction sites within MS‐AFLP fragments that exhibit differential CG methylation (Fulneček & Kovařík, 2014). Therefore, we combined type II variation and type III variation to represent the presence of DNA methylation in any context. Throughout this manuscript, we use “locus” to indicate a specific fragment size in the AFLP and MS‐AFLP results. We use “haplotype” to indicate the binary variable positions (dominant genotypes) for each individual's collection of AFLP loci, and “epigenotype” to indicate the collection of binary variable positions of MS‐AFLP loci.

### 2.4 Data analysis

To identify the number of different genetic groups represented in our collection independent of sampling location in our populations, we performed Bayesian clustering of the genetic data only using Structure v.2.3.4 (Falush, Stephens, & Pritchard, 2003, 2007; Hubisz, Falush, Stephens, & Pritchard, 2009; Pritchard, Stephens, & Donnelly, 2000). Our previous work has shown population structure within native *S. alterniflora* populations (Foust et al., 2016; Richards et al., 2004). Although we designed our sampling to avoid subpopulation structure in this study by only sampling near the leading edge of the marsh, we tested for the possibility of finding more populations than expected. We tested ten populations (k = 1–10) with ten independent runs at each k. We performed analyses with 50,000 burn‐in sweeps and 1,000,000 postburn‐in sweeps, assuming admixture and without including sample location, or any geographic information as priors in the analysis. We estimated the number of clusters represented by the data using Evanno's delta K (Evanno, Regnaut, & Goudet, 2005).

We used GenAlEx version 6.41 (Peakall & Smouse, 2012) to estimate the haplotype and epigenotype diversity (*h‐*AFLP and *h‐*MS‐AFLP). We also used GenAlEx to calculate estimates of genetic differentiation over all AFLP and MS‐AFLP loci with a hierarchical AMOVA, nesting study sites within oil exposure to compare genetic variation among oil‐contaminated and uncontaminated sites (Φ_RT_), among sites within contamination level (Φ_PR_), and within sites (Φ_PT_). We also used GenAlEx to conduct a locus‐by‐locus AMOVA to characterize genetic and epigenetic differentiation at each locus, using the same hierarchical design. Finally, we performed pairwise AMOVA comparisons to determine which populations were differentiated. For all AMOVA analyses, we used 9,999 permutations to estimate statistical significance and adjusted for multiple comparisons using the sequential Bonferroni method whenever multiple tests were performed.

In addition to AMOVA, we tested for the effect of oil on AFLP and MS‐AFLP multilocus marker profiles via permutational multivariate analysis of variance (perMANOVA), which allows for comparison of nested terms within hierarchical experimental design. Using the Adonis function within the Vegan package of R (Oksanen et al., 2017), we derived *p*‐values based on 9,999 permutations within populations using the following formula: Adonis (AFLP genetic distance matrix~ oil exposure, strata = population, permutations = 10,000). In each analysis, variation in marker profiles was represented by the Euclidean distance matrices as calculated from the binary AFLP and MS‐AFLP methylation data (with interpolation of missing values) generated by GenAlEx 6.41. We also used the RDA function within the Vegan package of R (Oksanen et al., 2017) to conduct a partial redundancy analysis of the relationship between contamination level (presence or absence) and MS‐AFLP, while removing the effects of AFLP. We used the following formula: RDA [x ~ y + z] where x = the Euclidean epigenetic distance matrix generated by GenAlEx, y = site condition (presence or absence of oil), and z = the Euclidean genetic distance matrix generated by GenAlEx. To create the site condition matrix, we used zero to indicate uncontaminated sites and one to indicate contaminated sites. This strategy makes the assumption that differences between contaminated and uncontaminated populations will be essentially the same magnitude regardless of individual population differences.

## 3 RESULTS

### 3.1 Genetic diversity and structure

A power analysis indicated that we could detect an effect of oil contamination among seven groups using our sample sizes (Fitzpatrick, 2009), and previous work reports population differentiation in hierarchical analyses is detectable with as few as five individuals per population (Nelson & Anderson, 2013). We found 71 polymorphic loci, which is well above the minimum of 30 markers reported in previous work to be required to detect significant patterns of differentiation (Nelson & Anderson, 2013). Of these loci, six were present or absent in only one sample. We ran these analyses with and without including these single‐variable loci and found no substantial differences in the results. The data presented here are based on the complete set of 71 polymorphic loci. Although a modest data set, our markers identified that genetic diversity was high (*h‐*AFLP ranged from 0.103 to 0.206), and 55 of 56 individuals displayed a unique genotype. There was no difference in genotype diversity between contaminated and uncontaminated sites (*p *=* *.262). Bayesian clustering identified two genetic groups (ΔK = 1,517.81); however, these groups did not clearly reflect either differentiation by oil contamination or geographic separation (Figure 1).

**Figure 3:**
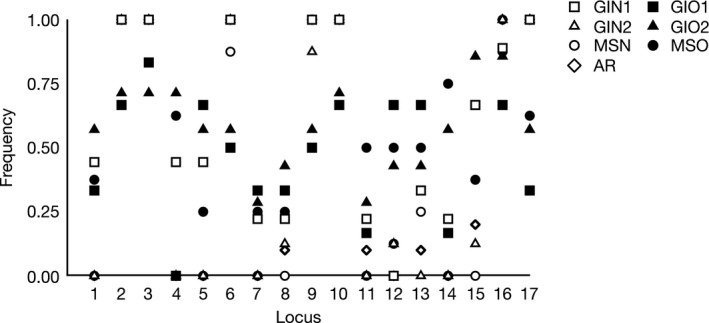
Frequencies of genetic loci significantly correlated to oil contamination across seven populations in locus‐by‐locus analysis. Contaminated sites are shown in closed shapes and uncontaminated sites in open shapes

Hierarchical AMOVA revealed significant variation between contaminated and unaffected populations (explaining 6% of the genetic variance), and among populations within site type (explaining 16% of the genetic variance; Table 3), as well as most (66%) pairwise comparisons between sites (Table 4), indicating the presence of population structure between contamination types and among populations. These results were supported by perMANOVA, which showed a significant effect of oil contamination on multilocus genetic marker profiles (*F* = 0.092, *p = *.017). Locus‐by‐locus AMOVA revealed 17 loci that varied significantly between oil‐contaminated and unaffected sites (Figure 3).

### 3.2 Epigenetic diversity and structure

We found 39 polymorphic epigenetic loci from 71 observed. Of these loci, seven were present or absent in only one sample. We ran these analyses with and without including these single‐variable loci and found no substantial differences in the results. The data presented here are based on the complete set of 39 polymorphic loci. Epigenotype diversity was high (*h‐*MSAFLP ranged from 0.089 to 0.222), and each individual displayed a unique epigenotype. Like the estimates for genetic patterns, there was no difference in epigenotype diversity between affected and unaffected sites (*p *=* *.993), and as in our previous studies of *S. alterniflora* (Foust et al., 2016), *h‐*MS‐AFLP tended to be lower than *h‐*AFLP (Table 2).

**Table 2:** Mean AFLP haplotype and MS‐AFLP epigenotype diversity (*h*) and percent polymorphic loci by site (%P), based on 71 AFLP and 39 MS‐AFLP loci

Population	AFLP		MS‐AFLP	
	*h*‐ (SE)	% P	*h*‐ (SE)	% P
Oil‐contaminated				
GIO1	0.216 (0.031)	40.85	0.179 (0.037)	41.03
GIO2	0.246 (0.035)	42.25	0.185 (0.037)	43.59
MSO	0.216 (0.028)	50.70	0.161 (0.030)	41.03
Unaffected				
GIN1	0.246 (0.027)	57.75	0.226 (0.031)	66.67
GIN2	0.190 (0.022)	57.75	0.152 (0.031)	46.15
MSN	0.138 (0.020)	50.70	0.204 (0.037)	48.72
AR	0.103 (0.021)	28.17	0.132 (0.033)	33.33

**Figure 4:**
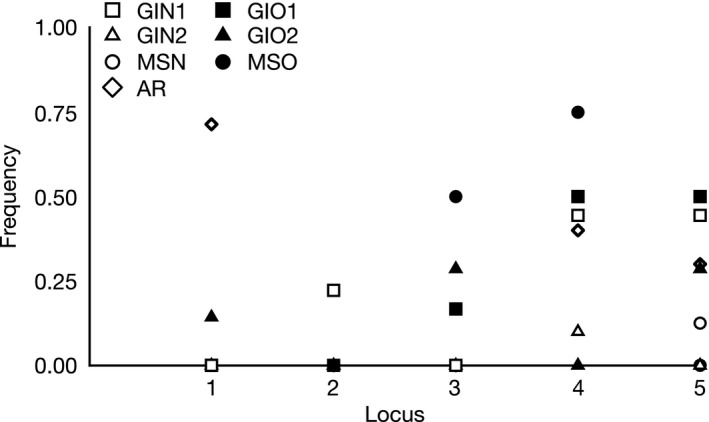
Frequencies of epigenetic loci significantly correlated to oil contamination across seven sites in locus‐by‐locus analysis. Contaminated sites are shown in closed shapes and uncontaminated sites in open shapes

Hierarchical AMOVA failed to detect a significant effect of oil contamination on epigenetic differentiation, but among populations within site type explained 7% of the epigenetic variance (Table 3), and 38% of the pairwise comparisons between sites were significant (Table 4). The lack of an effect of oil contamination on overall epigenetic variation was supported by perMANOVA (*F* = 0.373, *p = *.815) and redundancy analysis (*F* = 6.7269, *p = *.22). Locus‐by‐locus AMOVA revealed five loci were significantly differentiated between oil‐contaminated and unaffected sites (Figure 4).

**Table 3:** Summary of hierarchical AMOVA for AFLP and MS‐AFLP data sets among site type (Φ_RT_), among populations within site type (Φ_PR_), and within populations (Φ_PT_). Φ‐statistics were calculated using 9,999 permutations

	Genetic			Epigenetic		
	Φ‐statistics	% variation	*df*	Φ‐statistics	% variation	*df*
Among site type	0.056*	6	1	0.017^NS^	1	1
Among populations within site type	0.168***	16	5	0.076***	7	5
Within subpopulations	0.215***	78	49	0.071**	92	49

*df*, degrees of freedom.

*p ≤* *.05, **p ≤* *.01, ***p ≤* *.001

^NS^nonsignificant following sequential Bonferroni correction.

**Table 4:** Pairwise Φ_PT_ comparisons of variation among study sites. Epigenetic comparisons are shown above the diagonal, genetic below

	Unaffected sites				Oil‐contaminated sites		
	GIN1	GIN2	MSN	AR	GIO1	GIO2	MSO
GIN1		0.007	0.012	**0.124**	0.000	0.064	0.042
GIN2	**0.193**		0.013	0.205	0.024	0.039	0.133
MSN	**0.191**	0.150		**0.145**	0.018	0.032	0.077
AR	**0.189**	0.067	**0.180**		**0.166**	**0.160**	**0.208**
GIO1	0.123	**0.216**	**0.167**	**0.225**		0.000	0.137
GIO2	0.067	**0.261**	**0.247**	**0.319**	0.062		**0.159**
MSO	0.038	**0.197**	**0.190**	**0.217**	0.119	0.078	

Statistical significance after sequential Bonferroni correction denoted by bolded numbers.

## 4 DISCUSSION

Following the *Deepwater Horizon* oil spill in 2010, we sampled contaminated and uncontaminated populations of *S. alterniflora* along the coast of the Gulf of Mexico in populations that had experienced heavy oiling and complete aboveground dieback. Despite reports of full recovery of aboveground biomass and stem density in heavily oiled populations after 18 months (Lin et al., 2016), our hierarchical AMOVA returned evidence of genetic differentiation among oil‐contaminated and noncontaminated populations. However, we did not find evidence of decreased genetic diversity in contaminated populations, as nearly all individuals displayed a unique genotype in both contaminated and noncontaminated sites. These findings are consistent with other genetic surveys of *S. alterniflora* (Foust et al., 2016; Hughes & Lotterhos, 2014; Richards et al., 2004), which also show high levels of genetic variation. With our small sample size (*n* < 10 at most sites), it is possible that we were unable to capture a change in genetic diversity among populations if one occurred in response to the oil spill.

In addition, we found no evidence of epigenetic differentiation over all loci between oil‐contaminated and uncontaminated populations, but five loci showed epigenetic differentiation due to oil exposure in the locus‐by‐locus analysis. Further study is required to determine whether these loci are indicative of a regulatory response acting in concert with a few, but important epigenetic loci. However, redundancy analysis shows that overall patterns of methylation were not significantly correlated with oil exposure when controlling for the effects of genetic variation, which suggests that patterns of DNA methylation are explained almost entirely by genetic effects. Although we did expect to find epigenetic differentiation due to oil presence, it is possible either that oil did not induce any epigenetic changes between the population types, or that any existing epigenetic signature was too labile or too weak to be detected given the high epigenetic variation between individuals at our sites. Alternatively, our MS‐AFLP may provide too few, anonymous markers to quantify epigenetic differentiation, and our small sample size may not have sufficient power to detect effects of rare epigenetic alleles or weak signatures of epigenetic change among the genetically differentiated populations. Many previous studies of epigenetic variation have taken advantage of low genetic diversity in natural systems to more clearly delineate population epigenetic effects (e.g., Gao, Geng, Li, Chen, & Yang, 2010; Richards et al., 2012). However, *S. alterniflora* is an outcrossing, wind‐pollinated grass with extremely high genetic diversity (Foust et al., 2016; Hughes & Lotterhos, 2014; Richards et al., 2004). These high levels of genetic polymorphism make it more difficult to partition epigenetic structure due to increased statistical noise and genetic‐dependent effects, particularly using anonymous genetic markers such as AFLP (but see e.g., Foust et al., 2016).

### 4.1 Genetic and epigenetic response to pollution

Human‐mediated environmental impacts have been well documented as potential evolutionary drivers of population differentiation. A classic example is the rapid phenotypic change experienced by the peppered moth as a result of coal pollution (Kettlewell, 1958), which was recently explained by the activity of transposable elements that alter its development (van't Hof et al., 2016). Several studies also describe molecular differentiation in marine organisms across the eastern coast of the United States in response to aquatic pollution (Chapman et al., 2011; Whitehead et al., 2012; Williams & Oleksiak, 2008). For example, populations of Atlantic killifish (*Fundulus heteroclitus*) in severely polluted habitats show broad genetic differentiation, including an allelic variant of cytochrome CYP1A (Williams & Oleksiak, 2008, 2011), which is correlated with changes in gill morphology. Populations of the related Gulf killifish (*F. grandis*) in the Gulf of Mexico also showed differential expression of CYP1A among affected and unaffected populations following the *Deepwater Horizon* oil spill (Whitehead et al., 2012). Together, these studies highlight the role of anthropogenic stress in selection, adaptation, and divergence (Hoffmann & Sgrò, 2011; Lande, 1998). Despite previous literature suggesting that *S. alterniflora* is robust to heavy oil exposure, we found a signature of genetic differentiation between oil‐exposed and unexposed populations. These results suggest at least some mortality in oil‐exposed populations, consistent with findings of initial losses in live belowground biomass (Lin et al., 2016). By examining the genetic and epigenetic composition of marshes after the *DWH* oil spill, our study adds to the growing number of ecological and evolutionary genomics studies describing population‐level response to pollution.

Populations in coastal habitats, and salt marshes in particular, have long been models for phenotypic differentiation across natural environmental gradients (Schmidt & Rand, 1999; Schmidt et al., 2008), and we expected to detect population‐level differentiation of DNA methylation in response to oil contamination as well (Foust et al., 2016; Richards et al., 2012). The idea that epigenetic mechanisms can contribute to population differentiation as a source of heritable phenotypic variation has been challenged in recent literature (Laland et al., 2014; Wibowo et al., 2016). However, DNA methylation has been posited as a mechanism of phenotypic plasticity as well as a marker of stress response, and a number of studies have found a relationship between epigenetic variation and environment in support of this hypothesis (Herman & Sultan, 2016; Jablonka & Raz, 2009; Verhoeven, Jansen, et al., 2010; Verhoeven, Van Dijk, et al. 2010). Environmental stressors can induce variation in DNA methylation and in some cases, these environmentally induced methylation patterns can be inherited (Herrera & Bazaga, 2010, 2011; Verhoeven, Jansen, et al., 2010; Verhoeven, Van Dijk, et al. 2010), suggesting the potential for a signature of environmental response that is partially distinct from genetic variation.

Although we found high levels of epigenetic variation among individuals within and among populations, we failed to detect epigenetic differentiation in response to oil contamination. Our previous work showed a weak correlation between environmental conditions and epigenetic variation in *S. alterniflora* in a Georgia salt marsh (Foust et al., 2016). However, these data were collected from relatively protected habitat, and populations from this area are unlikely to have been exposed to a stress as severe as the *DWH* oil spill, which resulted in total aboveground dieback, and reduction by approximately 84%–95% of belowground biomass of the leading 5–10 meters of *S. alterniflora* in heavily oiled Gulf of Mexico marshes (Lin et al., 2016; Silliman et al., 2012). This impact may be far beyond what is normally experienced by *S. alterniflora* including natural disturbance events (Pennings & Bertness, 2001).

Epigenetic mechanisms of response, such as DNA methylation, are expected to be evolutionarily favorable when the periodicity of a stressor is short (Lachmann & Jablonka, 1996), such as cyclic patterns of rainfall, nutrient flows, and salinity that cause the zonation patterns observed among salt marsh plants (Pennings & Bertness, 2001). In contrast, the *Deepwater Horizon* oil spill may have acted as a single, discrete event that changed the makeup of the extensive genetic variation present in *S. alterniflora* rather than inducing a plastic or regulatory response that could be captured by assaying DNA methylation.

As studies of epigenetic variation in natural populations move away from quantifying the amount of standing genetic and epigenetic variation in natural populations to describing the role of that variation and the relative contribution of genetic and epigenetic variation to population differentiation, more precise sampling techniques and analyses will be needed. In future studies, a reduced‐representation bisulfite sequencing approach would allow the direct comparison of genetic and epigenetic data sets, and at a much finer scale, with substantially increased statistical power to detect epigenetic differences between populations (van Gurp et al., 2016; Robertson & Richards, 2015; Schrey et al., 2013; Trucchi et al., 2016). In addition, sequencing‐based methods provide an increased ability to disentangle the relationship of methylation variation and gene function when fragments overlap with the promoters or coding regions of genes. By increasing the number of loci surveyed, future studies may better identify the environmental conditions under which genetic or epigenetic variation is associated with environmental cues (Robertson & Richards, 2015).

## DATA ACCESSIBILITY

Data available from the Dryad Digital Repository: https://doi.org/10.5061/dryad.pf0s5.
